# Transcriptome Analysis of Antennal Chemosensory Genes in *Curculio Dieckmanni* Faust. (Coleoptera: Curculionidae)

**DOI:** 10.3389/fphys.2022.896793

**Published:** 2022-05-09

**Authors:** Xiaoqian Ma, Xinming Lu, Ping Zhang, Xun Deng, Jianyang Bai, Zhe Xu, Jian Diao, Hongyang Pang, Qi Wang, Hongying Zhao, Wei Ma, Ling Ma

**Affiliations:** ^1^ College of Forestry, Northeast Forestry University, Harbin, China; ^2^ Forest Protection Research Institute, HeiLongJiang Academy of Forestry, Harbin, China; ^3^ Mudanjiang Branch, HeiLongJiang Academy of Forestry, Mudanjiang, China; ^4^ College of Medicine, Heilongjiang University of Chinese Medicine, Harbin, China

**Keywords:** curculionoidea, antennal transcriptome, chemosensory genes, control strategies, curculio dieckmanni

## Abstract

The olfactory system plays a key role in regulating insect behaviors, such as locating host plants, spawning sites, and mating partners and avoiding predators. Chemosensory genes are required for olfactory recognition in insects. *Curculio dieckmanni* Faust. (Coleoptera: Curculionidae) damages hazelnuts and causes severe economic losses. There are no effective control measures, but understanding the olfaction mechanisms of this insect could lead to a new approach for population management. However, the genes that perform chemosensory functions in *C. dieckmanni* are still unclear. Using high-throughput sequencing, we assembled the antennal transcriptome of *C. dieckmanni* and annotated the major chemosensory gene families. Of the chemosensory gene families, we found 23 odorant-binding proteins, 15 chemosensory proteins, 2 sensory neuron membrane proteins, 15 odorant receptors, 23 ionotropic receptors, and nine gustatory receptors. Using Blast sequence alignment and phylogenetic analysis, the sequences of these proteins were identified. Male- and female-specific chemosensory genes involved in odorant detection and recognition were validated by qRT-PCR. Among the chemosensory genes, we found significant differences in the expression of CdieOBP8, CdieOBP9, CdieOBP19, CdieOBP20, CdieOBP21, CdieCSP15, CdieOR13, and CdieOR15 between adult male and female *C. dieckmanni*. A total of 87 expressed chemosensory proteins were found in *C. dieckmanni*. Investigating these proteins will help reveal the molecular mechanism of odorant recognition in *C. dieckmanni* and may aid the development of novel control strategies for this species.

## Introduction

*Curculio dieckmanni* Faust. is a pest beetle that infests hazelnut (*Corylus heterophylla* Fisch.) crops and is common in hazelnut orchards of China. The adults lay eggs in hazelnut trees, and the larvae feed on, and damage, the nut causing significant economic losses (Xiao, 1992; Zhang et al., 2021). Because of the damage caused by this pest, the State Forestry Administration of China listed *C. dieckmanni* as a national harmful pest for forestry in 2013 and initiated measures to reduce the economic impact that it causes in hazelnut orchards (Announcement of the State Forestry Administration of China, 2013). *C. dieckmanni* has overlapping generations and nonsynchronous life cycles, and it is presently controlled by pesticide applications. However, long-term use of broad-spectrum pesticides can result in toxic residues and lead to pest resistance (Ahmad et al., 2002; Chai and Lee, 2010; Downes, et al., 2016). In addition, pesticide residues in hazelnuts can be detrimental to human health and the environment. Therefore, it is crucial to develop novel control methods that are safer and less ecologically disruptive.

The olfactory chemosensory system performs important functions in the life stages of insects because it detects olfactory information carried by odorant molecules related to specific behaviors such as congregating, locating reproductive sites and mates, foraging, and avoiding predators (Field et al., 2000; Smadja and Butlin, 2009; Leal, 2013; Tian et al., 2018; Xiao et al., 2019). When lipophilic odorant molecules enter the lymph through micropores on chemosensory sensilla located in the antennal epidermis, they are detected by olfactory neurons (ORNs). The chemical information signals will be transformed into electrophysiological information signals. These physiological signals are transduced by ORNs to the central nervous system of the insect brain, where the information is processed to trigger specific physiological and behavioral responses through neurons (Vosshall et al., 1999; Vogt et al., 2009). In the process of odorant molecule detection, odorant molecules are first bound and transported by odorant-binding proteins (OBPs) and chemosensory proteins (CSPs) in sensory lymph fluid, which is the first step in the transport of hydrophobic odorants in olfactory recognition (Dippel, 2014; Keil and Steinbrecht, 1984; Wanner et al., 2005; Pelosi et al., 2006; Guo et al., 2011; Pelosi et al., 2014; Pelosi et al., 2018; Xiao et al., 2019; Liu et al., 2020). Then, these molecules are detected by odorant receptors protein (ORs) or ionotropic receptors (IRs) expressed on the olfactory neuron membrane (Benton et al., 2009; Croset et al., 2010; Abuin et al., 2011). ORs are divided into co-receptors (ORco) and common odorant receptors. ORco is highly conserved across species and can enhance the ability of specific common odorant receptors to bind to odorant molecules (Jacquin-Joly and Merlin, 2004; Benton et al., 2006; Sato and Touhara 2009). In coleopteran insects, ORs are divided into seven monophyletic subfamilies, called nine groups, according to their amino acid sequences (Mitchell and Andersson, 2020). Additionally, gustatory receptors (GRs) and sensory neuron membrane proteins (SNMPs) are also involved. GRs mainly detect sugars, bitter flavors, pheromones, CO_2_, or contact stimuli. They perform important functions in host-seeking and are not limited to gustatory functions (Clyne, et al., 2000; Park and Kwon, 2011; Mang et al., 2016). Sensory neuron membrane proteins (SNMPs) contain two transmembrane domains, which play an essential role in identifying and detecting pheromones (Clyne et al., 1999; Mang et al., 2016).

Early research on chemosensory genes in coleopteran insects has focused mainly on OBPs (Vogt and Riddiford, 1981). The use of OBPs to detect pheromones was subsequently recorded in scarabs (2001). In Coleoptera, the earliest study of receptors came from the wasp co-receptor ORco, followed by the first genome study of the full set of chemosensory genes in coleopteran model insects (Krieger et al., 2003). The most complete study of chemosensory genes was conducted in other species of Coleoptera (Engsontia et al., 2008; Nichols and Vogt, 2008; Richards et al., 2008). In recent years, most studies have focused on chemosensory gene sequences and gene expression, while studies on functional characterization are relatively rare. ORs have a key role in pheromone detection. The ItpyOR46 and ItpyOR49 of *I. typographus* were specifically reactive with pheromone compounds (S)-ipsenol and (R)-(-)-ipsdienol, respectively, and phylogenetic analysis was performed in the OR subfamily of coleopteran group 7, which is widely present in the weevil family (Yuvaraj et al., 2021). McOr3 and McOr20 of *Megacyllene caryae* are sensitive to the male-secreted pheromones (S)-2-methyl-1-butanol and (2S, 3R)-2,3-hexanediol, respectively (Mitchell et al., 2012). The red palm weevil is rendered incapable of recognizing the male-produced pheromones (4RS, 5RS)-4-methylnonan-5-ol and 4 (RS)-methylnonan-5-one by silencing RferOR1 (Antony et al., 2021).

With the development of next generation sequencing (NGS) in insects, many chemosensory genes have been sequenced and verified by genomic and transcriptomic data. NGS has improved our knowledge. Despite substantial progress in deciphering these chemical communication systems in insects, the study of chemosensory genes in Coleoptera is challenging because of the profound diversity of Coleoptera. There are nearly 200 families in Coleoptera, and the annotation of chemosensory genes mainly revolves around several genera in five families (Cerambycidae, Chrysomelidae, Curculionidae, Scarabaeidae, and Tenebrionidae) (Mckenna et al., 2019). In addition to the coleopteran model insect *T. castaneum*, the genomes of *Leptinotarsa decemlineata*, *Anoplophora glabripennis*, *Agrilus planipennis*, and *Dendroctonus ponderosae* have been established (Mckenna et al., 2016; Mitchell et al., 2017; Schoville et al., 2018; Andersson et al., 2019; Mitchell et al., 2020). These genomes help to establish the phylogenetic framework of coleopteran chemosensory genes. The annotations of the remaining chemosensory genes are derived from the transcriptome, which is currently a handy and economical sequencing method suitable for the annotation of insect chemosensory genes. However, there is little information regarding the molecular mechanism underlying olfactory recognition in Curculionoidea. Very little research is available on the molecular mechanisms of olfactory recognition of *C. dieckmanni*. The Curculionidae (weevils) is one of the largest coleopteran families with more than 80,000 species described, some of which are economically important pests. Only the chemosensory gene families of a dozen Curculionidae species have been studied (Andersson et al., 2013; Bin et al., 2017; Paula et al., 2018; Andersson et al., 2019; Zhang et al., 2019; Antony et al., 2021; Hou et al., 2021; Yuvaraj et al., 2021). The olfactory genes of *C. dieckmanni* have not been studied yet, and their identification will help to illustrate the molecular mechanisms of olfactory recognition in this family.

In this work, the antennal transcriptomes of female and male adults of *C. dieckmanni* were sequenced on an Illumina HiSeqTM 4000 platform. After the transcriptome data were assembled and analyzed, we identified 23 OBPs, 15 CSPs, 2 SNMPs, 15 ORs, 23 IRs and 9 GRs. The expression patterns of chemosensory genes in the antennae of male and female adults were analyzed by reads per kilobase per million (RPKM) and the expression profiles of these genes were verified by qRT-PCR. In addition, phylogenetic trees were constructed to predict the evolutionary relationship between the chemosensory genes of *C. dieckmanni* and other Curculionoidea species. These results help validate the functions of olfactory genes and provide a foundation for a more detailed study of the molecular mechanisms of olfactory recognition in *C. dieckmanni*.

## Materials and Methods

### Rearing and Treatment of Experimental Insects

Adult *C. dieckmanni* were collected in a hazelnut orchard in the Sandao Forestry Farm, Mudanjiang City, Heilongjiang Province, China (129°39′02″E, 44°07′04″N). The insects were fed with hydroponic C. heterophylla shoots in a cage (80 cm × 40 cm × 40 cm). Males and females were separated and dissected under a dissecting microscope to collect the antennae, proboscis, head, legs, wings, prothorax, and abdomen. Tissue samples were separately stored in Axygen tubes and frozen at −80°C. A total of 200 pairs of antennae were dissected from female and male adults for transcriptome sequencing, and there were three biological replicates for female and males, including six samples (DM1, DM2, DM3, DF1, DF2, and DF3).

### RNA Quality Assessment

Total RNA was isolated from the antennae of female and male adults of *C. dieckmanni*, using a Trizol reagent kit (Invitrogen, Carlsbad, CA, United States) based on the manufacturer’s instructions. The integrity of RNA quality was assessed using an Agilent 2100 bioanalyzer (Agilent Technologies, Palo Alto, CA, United States) and examined by RNase free agarose gel electrophoresis.

### RNA Sequencing and Library Construction

After the total RNA examination was qualified, the mRNA was enriched with magnetic beads with Oligo (dT). We added fragmentation buffer to break the mRNA into short fragments. We then used mRNA as a template for amplification and added random hexamers for reverse transcription to synthesize the first strand cDNA. We added buffer, dNTPs, and reagents, such as DNA polymerase I and RNase H, to synthesize the second-strand cDNA and purified the double-stranded cDNA with AMPure XP beads. The purified double-stranded cDNA was end repaired, poly (A) was added, and the cDNA was connected to Illumina sequencing adapters. We used AMPure XP beads to select the size of the fragments. PCR amplification was performed, and the PCR products were purified with AMPure XP beads to obtain the final cDNA library. After the library was constructed, Qubit 2.0 was used to determine the concentration, and the library was diluted to 1.5 ng/μl. Agilent 2100 was used to detect the insert size of the library. To ensure the quality of the library, the qPCR method was used to accurately quantify the effective concentration of the library. Qualified libraries of female and male adult antennae were sequenced using Illumina HiSeqTM 4000 by Gene Denovo Biotechnology Co. (Guangzhou, China). All the raw sequence data were saved in the NCBI Sequence Read Archive (SRA) database under BioProject accession No. PRJNA769532.

### Do Novo Assembly, Functional Annotation, and Classification

To ensure analysis quality, the original data obtained by sequencing were converted into sequence data by base calling, which contains joints, duplicates, and low sequencing quality. The reads were filtered to avoid affecting sequence assembly and subsequent analysis. We removed reads containing adaptors, reads with a proportion of unknown nucleotides (N) greater than 10%, and reads with low quality (*q*-value ≤ 20) over 40% bases. After obtaining the clean reads, we calculated the Q20, Q30 and GC content of the clean reads, and used Trinity to assemble the transcriptome from scratch. Trinity is a short reads assembly software (Grabherr et al., 2011).

The results of Trinity assembly were unigenes. To obtain the functional information of the unigenes, the unigene sequences were subjected to basic annotations, including protein function annotations, COG/KOG function annotations, pathway annotations and gene ontology (GO) annotations. For unigene annotation, at the E-value threshold of 1e-5, through BLASTx, all unigenes were compared in the NCBI non-redundant protein database (Nr), manually annotated, and reviewed in the Swiss-Prot protein database, the Kyoto Encyclopedia of Genes and Genomes (KEGG) database, and the Clusters of Orthologous Groups of proteins (COG/KOG) database. According to the best alignment results, unigene sequence directions were determined and protein function annotations were obtained. We used Blast2GO software for GO annotation and unigenes Nr result annotation (Conesa, et al., 2005). We selected the top 20 unigenes with the highest scores and no less than 33 High-scoring Segment Pair (HSPs) hits for Blast2GO analysis. WEGO software was used to classify the unigenes (Ye, et al., 2006).

### Chemosensory Gene Verification and Phylogenetic Analysis

The predicted conserved domains and open reading frames (ORFs) were manually validated for all candidate chemosensory genes (OBPs, CSPs, ORs, SNMPs, GRs, and IRs). Sequences with errors (insertions/gaps/deletions in homopolymer regions) were deleted. The putative N-terminal signal peptides of ligand-binding proteins (OBPs and CSPs) were searched using the SignalP v4.1 program. The transmembrane domains (TMDs) of ORs, IRs, and GRs were searched using TMHMM Server version 2.0. The translation of nucleotide sequences was performed using the Expasy program.

To validate the annotation of candidate chemosensory genes and the identification of homologous sequences, we conducted a phylogenetic analysis of *C. dieckmanni* including closely related Coleoptera species and other model insects. Insects selected for analysis were those whose chemosensory genes have been well studied (Supplementary Table S6). A multiple sequence alignment was performed using the MAFFT program, and the results were accessed using the Jalview software for editing and selecting the color scheme. Amino acid sequences of chemosensory genes of selected insects were obtained from the NCBI database, and Maximum likelihood (ML) phylogenetic trees were built based on the Jones-Taylor-Thornton (JTT) model in MEGA-X. Initial trees for the heuristic search were obtained automatically by applying Neighbor-Join and BioNJ algorithms to a matrix of pairwise distances estimated using the JTT model, then selecting the topology with the superior log likelihood value. The tree was drawn to scale with branch lengths measured in the number of substitutions per site (Jones et al., 1992; Kumar et al., 2018). Bootstrap support values were based on 100 replicates.

### Analysis of DEGs Based on RPKM

To identify chemosensory DEGs, we first used RPKM to estimate the expression of chemosensory genes in the antennae of male and female adults (Mortazavi et al., 2008). These genes were selected as reference for the expression profile of genes in the antennae of male and female adults. Gene expression levels were determined, according to RPKM values. In addition, DEGs were identified by Benjamini-Hochberg-corrected *p* values (Guo and Rao, 2008). As a key indicator, the false discovery rate (FDR) was used to screen in multiple hypothesis testing for gene selection. In comparison, genes with multiple changes ≥ 2 and FDR < 0.05 were identified as significant DEGs (Ran et al., 2020).

### qRT-PCR Analysis of Candidate Genes

The 31 genes in female and male transcriptomes were validated by qRT-PCR in different tissues and these genes, compared with all candidate genes, were highly expressed based on RPKM. Antennae (480) were collected from female and male adults. RNA was extracted using Trizol reagent and cDNA was synthesized using a PrimeScriptTM RT Reagent Kit with gDNA Eraser (TaKaRa, Shiga, Japan). We designed gene-specific primers using Primer3. The amplification efficiency was calculated for the genes and two candidate internal reference genes (actin and EF1-a). Based on the results, actin and EF1-a were selected as the reference genes for qRT-PCR. PCR was performed using Mx3000P QPCR (Agilent, United States) and ChamQ universal SYBR qPCR Master Mix (TaKaRa). The PCR program was as follows: 95°C for 30 s; 40 cycles of 95°C for 5 s and 60°C for 30 s; and 65–95°C at 0.5°C/5 s. A melting curve was generated. Each reaction had three independent biological replicates, and the relative gene expression level was obtained by the 2^−∆∆Ct^ method (Guo and Rao, 2008). The relative gene expression in antennae was analyzed using SPSS v19.0 software. An independent sample *t*-test was performed to compare the gene expression levels between male and female antennae. Prism v6.0 was used to generate bar graphs.

## Results

### Transcriptome Sequencing

A total of 42.5 Gb raw data were obtained after filtering. The transcriptomes of three female individuals yielded 49,095,002 (97.56%, DF1), 44,906,190 (97.60%, DF2), and 49,073,722 (97.47%, DF3) clean reads. The transcriptomes of three male individuals yielded 55,983,482 (97.52%, DM1), 49,406,426 (97.53%, DM2), and 41,989,468 (97.44%, DM3) clean reads. The Q20 value was >98%, and the Q30 value was >95% (Supplementary Table S1.1). The clean reads of six samples of females and males were assembled to yield a total of 43,060,197 unigenes. There were 44,024 unigenes longer than 200 bp (N50 = 1,861), including 42,971 unigenes from female samples (DF) and 43,190 unigenes from male samples (DM). The maximum length was 27,588 bp, the minimum length was 201 bp, and the average length was 978 bp (Supplementary Figure S1, Supplementary Table S1.2).

### Functional Annotation of Chemosensory Genes in *C. Dieckmanni*

The unigene sequences were aligned using BLASTx against the protein databases NR, SwissProt, KEGG, and KOG (*e*-value < 0.00001). The protein with the highest similarity to a given unigene was selected for protein function annotation, and 21,803 of 44,024 (49.52%) unigenes were successfully annotated. The numbers of reference gene sequences annotated in NR, Swiss-Prot, KEGG, and KOG were 21,733, 12,094, 7,502, and 11,403, respectively, (Supplementary Figure S2, Supplementary Table S1.2). The sequence with the best alignment (lowest *e*-value) of a unigene, which was selected as homologous in the NR database (if there was a tie, the first sequence was selected). The species of the homologous sequence was determined, and the number of homologous sequences of each species was counted. The sequences with high similarity to those in transcripts were searched in the NR protein database by comparison with BLASTx. The analysis revealed a large number of homologous genes in Coleoptera. The insect species with the highest homology (40.93%) was *Dendroctnous ponderosae*, followed by *Anoplophora glabripennis* (14.13%), *Tribolium castaneum* (5.01%), and *Lasius niger* (4.68%), which had 8,895, 3,071, 1,088, and 1,018 matched transcripts, respectively (Supplementary Figure S3).

Based on the NR annotation, the Blast2GO (Conesa et al., 2005) software was used to obtain the GO annotations of unigenes. The WEGO software (Ye et al., 2006) was used for GO functional classification of all unigenes to draw a whole picture of the gene functions of this species. These transcripts were assigned to three categories: molecular function (1,808 unigenes), biological process (3,675 unigenes), and cellular component (2,544 unigenes; Supplementary Figure S4). To accurately evaluate the annotation information, the transcripts were aligned to the KOG database, and a total of 11,403 transcripts were assigned to 25 functional categories (Supplementary Figure S5).

Of the chemosensory gene families, a total of 87 expressed chemosensory proteins were found in *C. dieckmanni*: 23 odorant-binding proteins, 15 chemosensory proteins, 2 sensory neuron membrane proteins, 15 odorant receptors, 23 ionotropic receptors, and nine gustatory receptors (Information for genes in Supplementary Tables S2.1–S2.6). The sequences of chemosensory genes identified in *C. dieckmanni* transcriptomes are shown in Supplementary Table S5.

### Candidate Genes Related to Transport Odorant Molecules

#### Odorant-Binding Proteins

**FIGURE 1:**
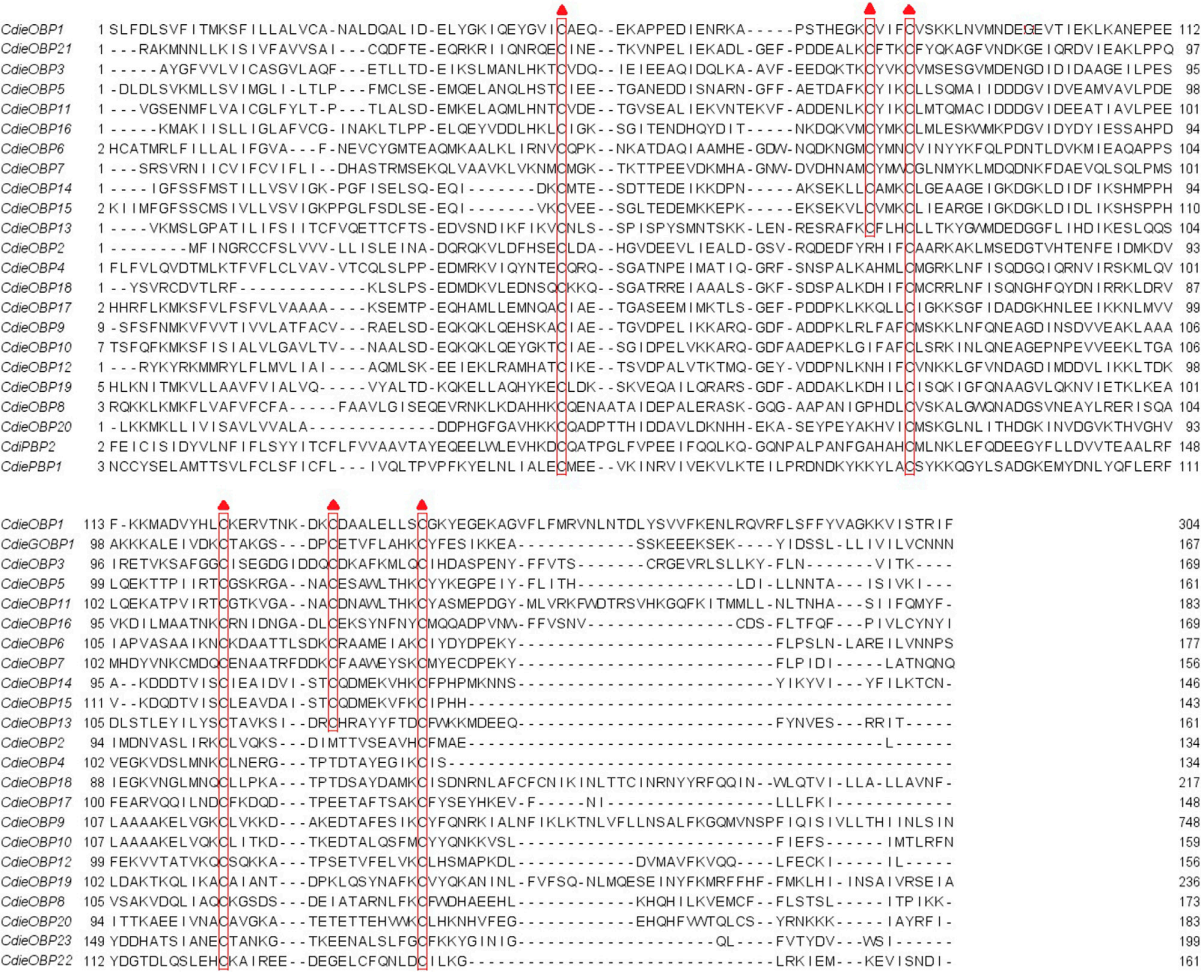
Alignment of candidate OBPs of C. dieckmanni. The highly conserved cysteine residues are marked with red triangles.

Twenty-three OBPs were obtained using BLASTx (Table S2.1). In the NCBI database, the homology of OBP sequences from *C. dieckmanni* and other Coleoptera species ranged from 37.40 to 78.05% (58.26% on average). In a further sequence analysis based on the number and location of cysteine (Figure 1), 11 OBPs (CdieOBP1, CdieOBP3, CdieOBP5, CdieOBP6, CdieOBP7, CdieOBP11, CdieOBP13, CdieOBP14, CdieOBP15, CdieOBP16, and CdieOBP21) were identified as typical “Classic OBP” subfamily with the sequence C1-X26-29-C2-X3-C3-X35-40-C4-X7-10-C5-X8-C6 (X is any amino acid), and 12 OBPs (CdieOBP2, CdieOBP4, CdieOBP8, CdieOBP9, CdieOBP10, CdieOBP12, CdieOBP17, CdieOBP18, CdieOBP19, CdieOBP20, CdieOBP22, and CdieOBP23) belonged to the “Minus-C OBP” subfamily with the sequence C1-X30-33-C2-X38-40-C3- X16-17-C4. The ORFs of 23 OBP genes ranged from 330 to 474 bp and encoded 110–157 amino acids. All genes except CdieOBP23 had complete ORFs, and six OBPs (CdieOBP9, CdieOBP11, CdieOBP18, CdieOBP19, CdieOBP21, and CdieOBP22) lacked signal peptides.

**FIGURE 2:**
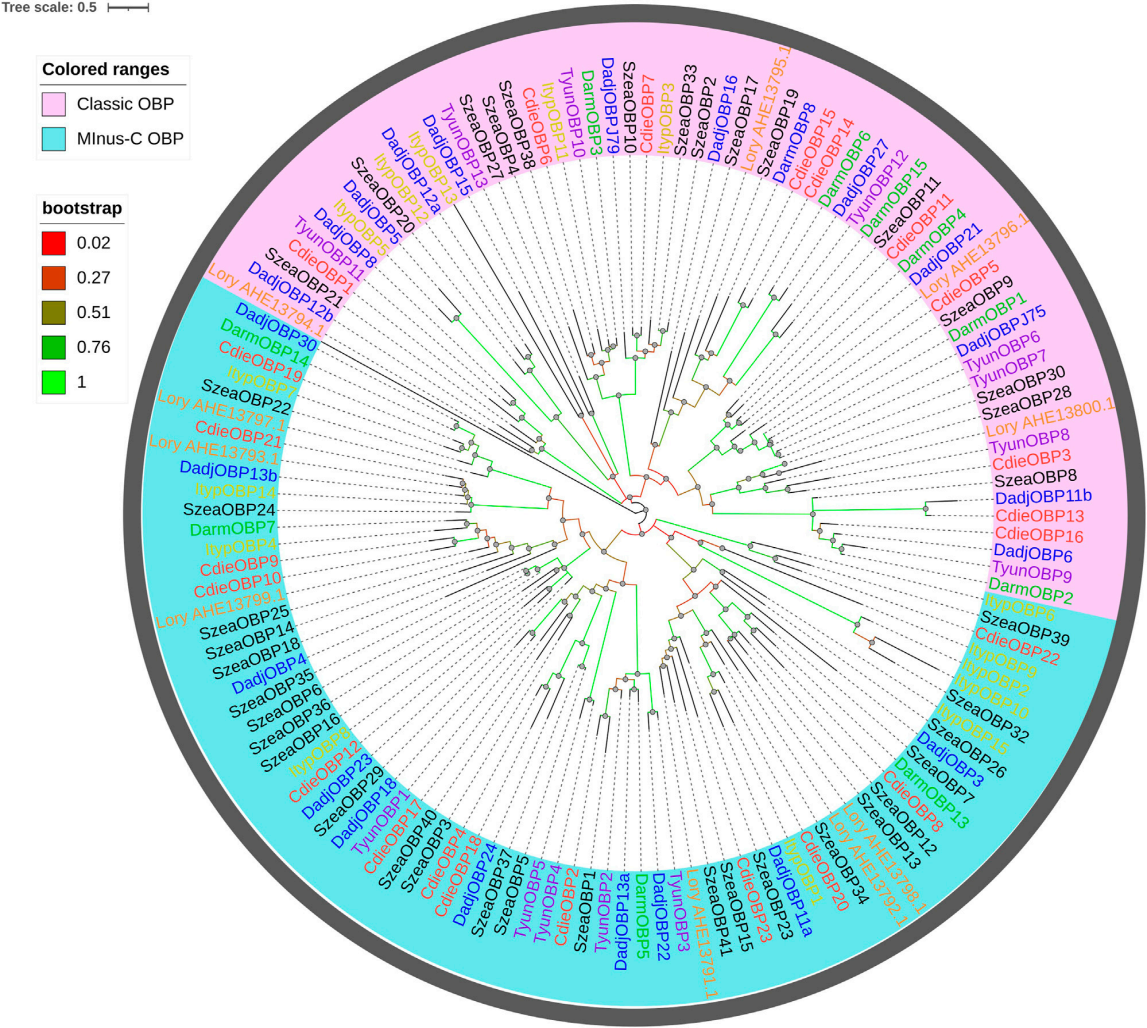
Maximum likelihood tree of OBPs in C. dieckmanni (Cdie, red), D. adjunctus (Dadj, blue), D. armandi (Darm, green), Ips typographus (Ityp, yellow), Lissorhoptrus oryzophilus (Lory, orange), S. zeamais (Szea, black), and T. yunnanensis (Tyun, purple). The scale bar represents 0.5 amino acid substitutions per site.

A phylogenetic tree was built using the 23 OBPs obtained from the transcriptomes and the OBPs from five Curculionoidea species (*Dendroctonus adjunctus, D. armandi, Lissorhoptrus oryzophilus, Sitophilus zeamais*, and *Tomicus yunnanensis*; Figure 2). These OBPs were highly conserved and were classified into “Minus-C OBP” or “Classic OBP” according to their functions. OBPs showed distinct patterns and did not cluster together. A homology matrix showed that adjacent branches had higher homology.

### Chemosensory Proteins

**FIGURE 3:**
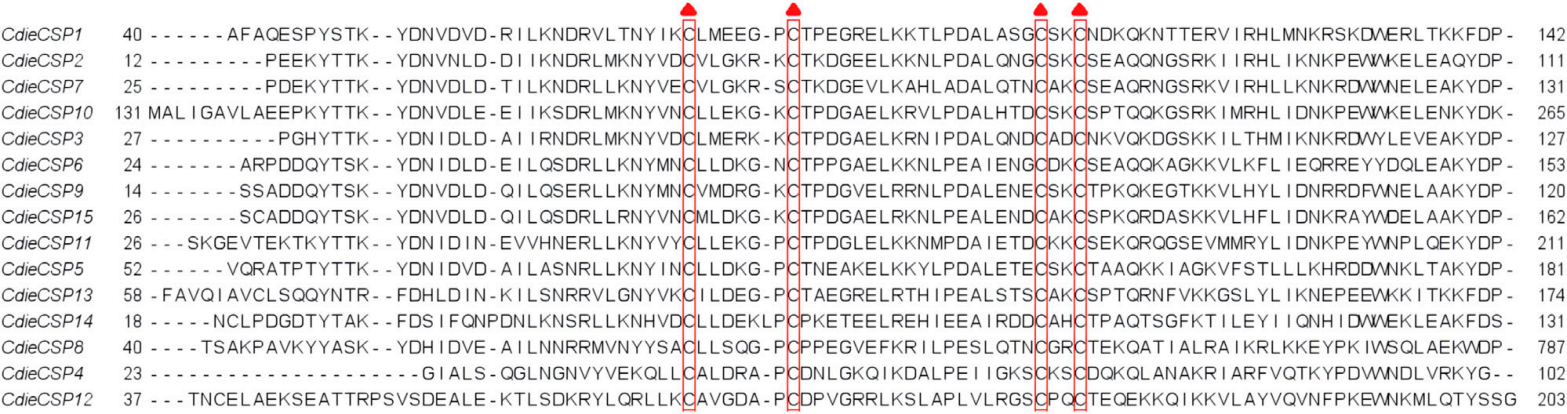
Alignment of candidate CSPs of C. dieckmanni. Each highly conserved cysteine residue is marked with a red triangle above it.

**FIGURE 4:**
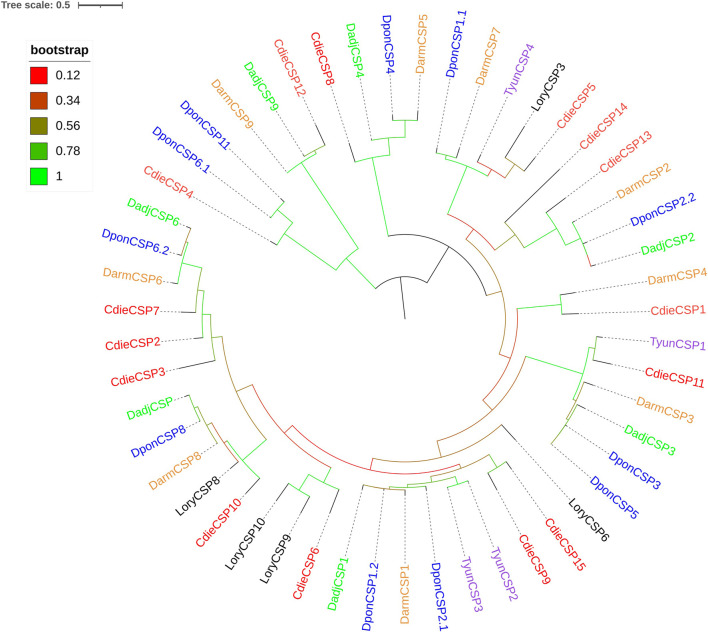
Maximum likelihood tree of CSPs in *C. dieckmanni* (Cdie, red), *Dendroctonus adjunctus* (Dadj, green), *Dendroctonus armandi* (Darm, orange), *Lissorhoptrus oryzophilus* (Lory, black), *Dendroctonus ponderosae* (Dpon, blue), and *Tomicus yunnanensis* (Tyun, purple). The scale bar represents 0.5 amino acid substitutions per site.

The analysis of transcriptome data revealed 15 CSPs, which had four conserved cysteine residues and the featured sequence C1-X6-7-C2-X18-C3-X2-C4 (X is any amino acid) of typical insect CSPs (Figure 3). The genes of 15 CSPs had ORFs ranging from 231 to 963 bp and encoding 76–320 amino acids, of which the genes of seven CSPs (CdieCSP1, CdieCSP2, CdieCSP4, CdieCSP5, CdieCSP6, CdieCSP7, and CdieCSP8) had incomplete ORFs, and eight CSPs (CdieCSP1, CdieCSP2, CdieCSP4, CdieCSP5, CdieCSP8, CdieCSP10, CdieCSP12, and CdieCSP13) lacked signal peptides (Supplementary Table S2.2). A phylogenetic tree was built using the 15 CSPs obtained from the transcriptomes and CSPs from five Curculionoidea species (*Dendroctonus adjunctus*, *D. armandi*, *Lissorhoptrus oryzophilu*s, *Dendroctonus ponderosae*, and *Tomicus yunnanensis*) (Figure 4).

### Sensory Neuron Membrane Proteins

**FIGURE 5:**
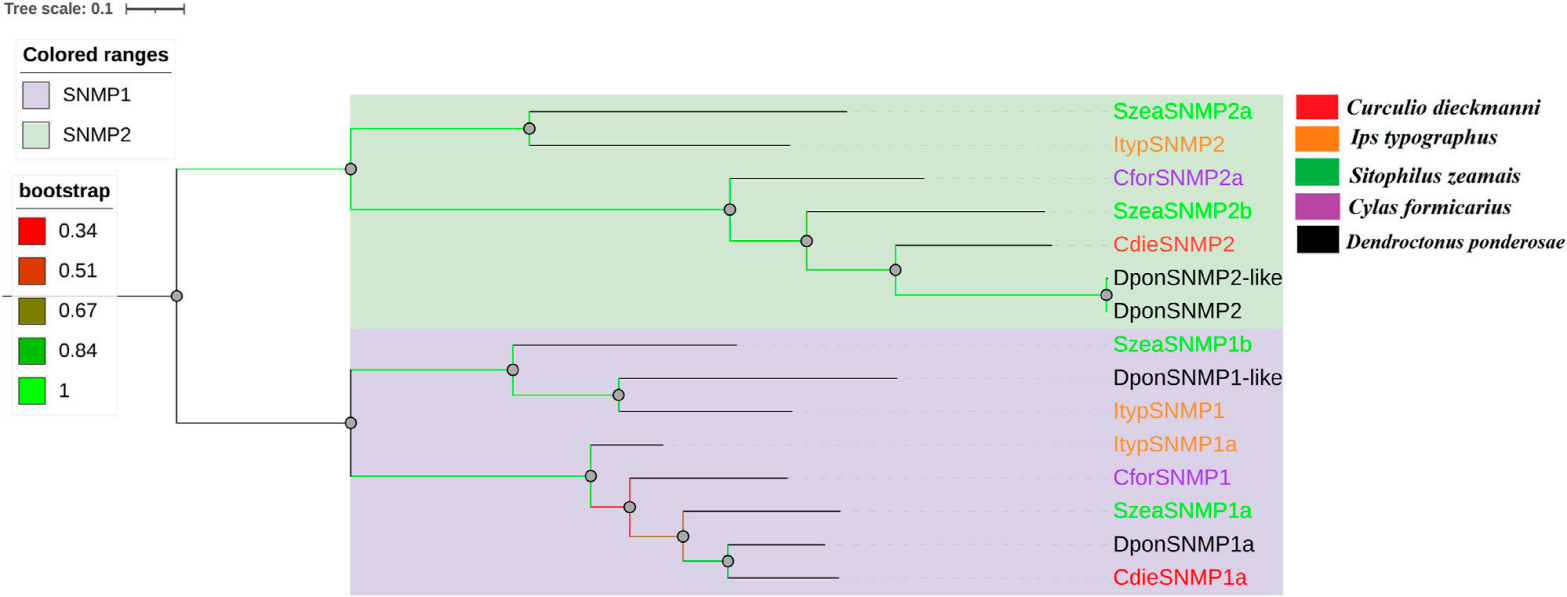
Maximum likelihood tree of SNMPs in C. dieckmanni (Cdie, red), *S. zeamais* (Szea, green), *Ips typographus* (Ityp, orange), *Cylas formicarius* (Cfor, purple), and *Dendroctonus ponderosae* (Dpon, black). The scale bar represents the 0.1 amino acid substitutions per site.

Genes with complete ORFs encoding CdieSNMP1 (SNMP1) and CdieSNMP2 (SNMP2) were identified based on BLASTx and cluster analysis. The ORF was 1,695 bp for CdieSNMP1 and 1,575 bp for CdieSNMP2, indicating that both were encoded by full-length genes. CdieSNMP1 and CdieSNMP1 had six and three transmembrane regions as well as 74.04 and 56.84% homology, respectively, (Supplementary Table S2.3). Phylogenetic analysis showed that CdieSNMP1 and CdieSNMP2 clustered with the SNMP1 and SNMP2 of other Curculionoidea species, respectively, and were most closely related to *D. ponderosae*, which confirmed the above results (Figure 5).

### Odorant Receptors

**FIGURE 6:**
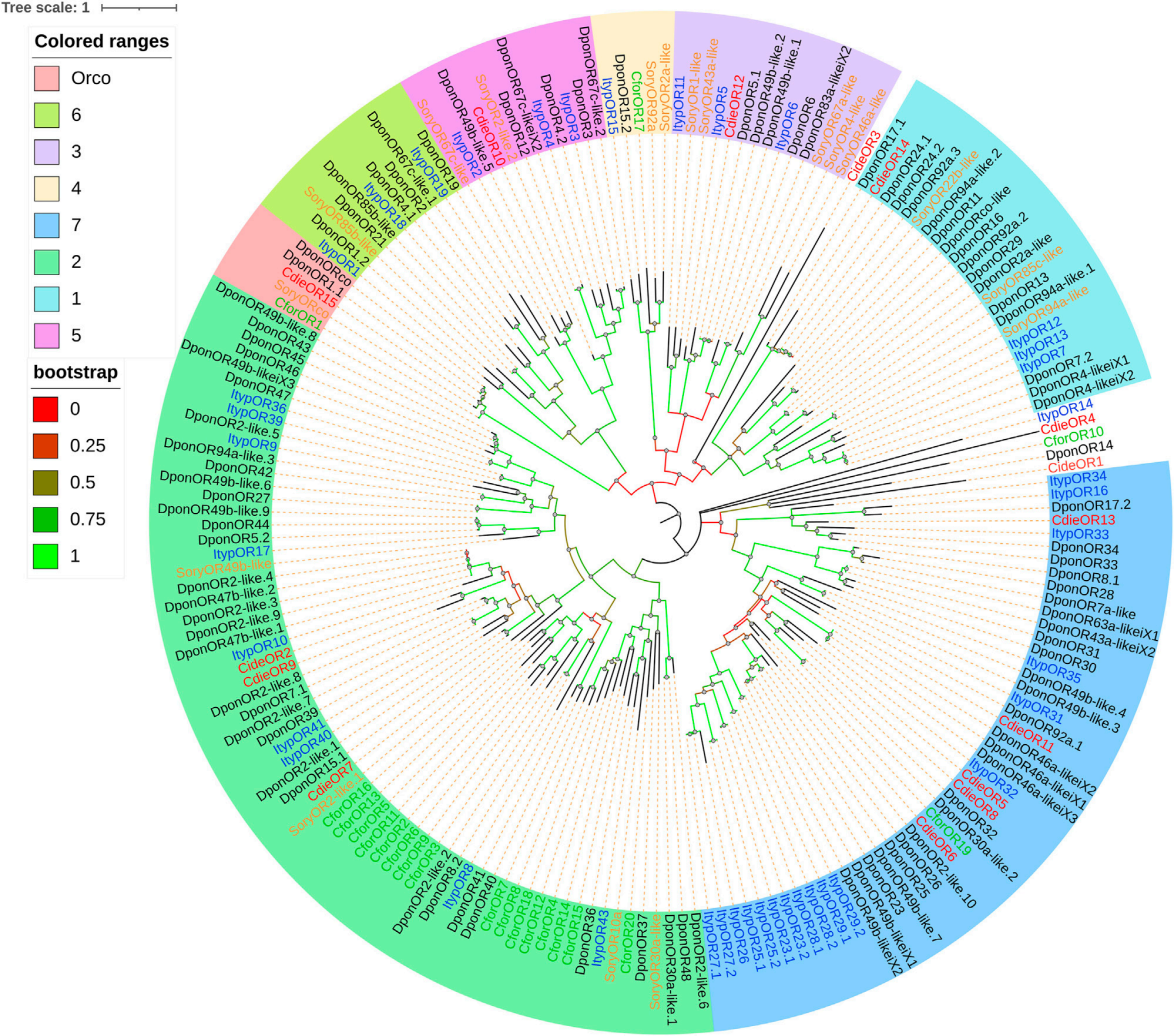
Maximum likelihood phylogenetic tree of candidate ORs in *C. dieckmanni* (Cdie, red), *Dendroctonus ponderosae* (Dpon, black), *Ips typographus* (Ityp, blue), *Cylas formicarius* (Cfor, green), and *Sitophilus oryzae* (Sory, orange). The scale bar represents 1 amino acid substitution per site.

A total of 15 ORs were identified in the analysis of transcriptome data, including one odorant receptor coreceptor (Orco). The alignment of ORs against the NR database showed 36.02–94.40% homology and 78–1,449 bp ORFs encoding 25–482 amino acids. Given the sequence characteristics of ORs, 1–9 transmembrane domains were predicted (Supplementary Table S2.4). A phylogenetic tree was established along with the ORs identified in other Curculionoidea species (*D. ponderosae*, *Ips typographus*, and *Sitophilus oryzae*; Figure 6). Based on the amino acid sequence analysis, the ORs from *C. dieckmanni* was contained in six groups of beetles (Bin et al., 2017; Mitchell et al., 2020). Among them, CdieOR15 clustered with the highly conserved ORco in a branch. CdieOR14 clustered in group 1, CdieOR12 clustered in group 3, CdieOR10 clustered in group 5, three candidate ORs (CdieOR2, CdieOR7, and CdieOR9) clustered in group 7, and four candidate ORs (CdieOR5, CdieOR6, CdieOR8, and CdieOR11) clustered in group 2.

### Ionotropic Receptors

**FIGURE 7:**
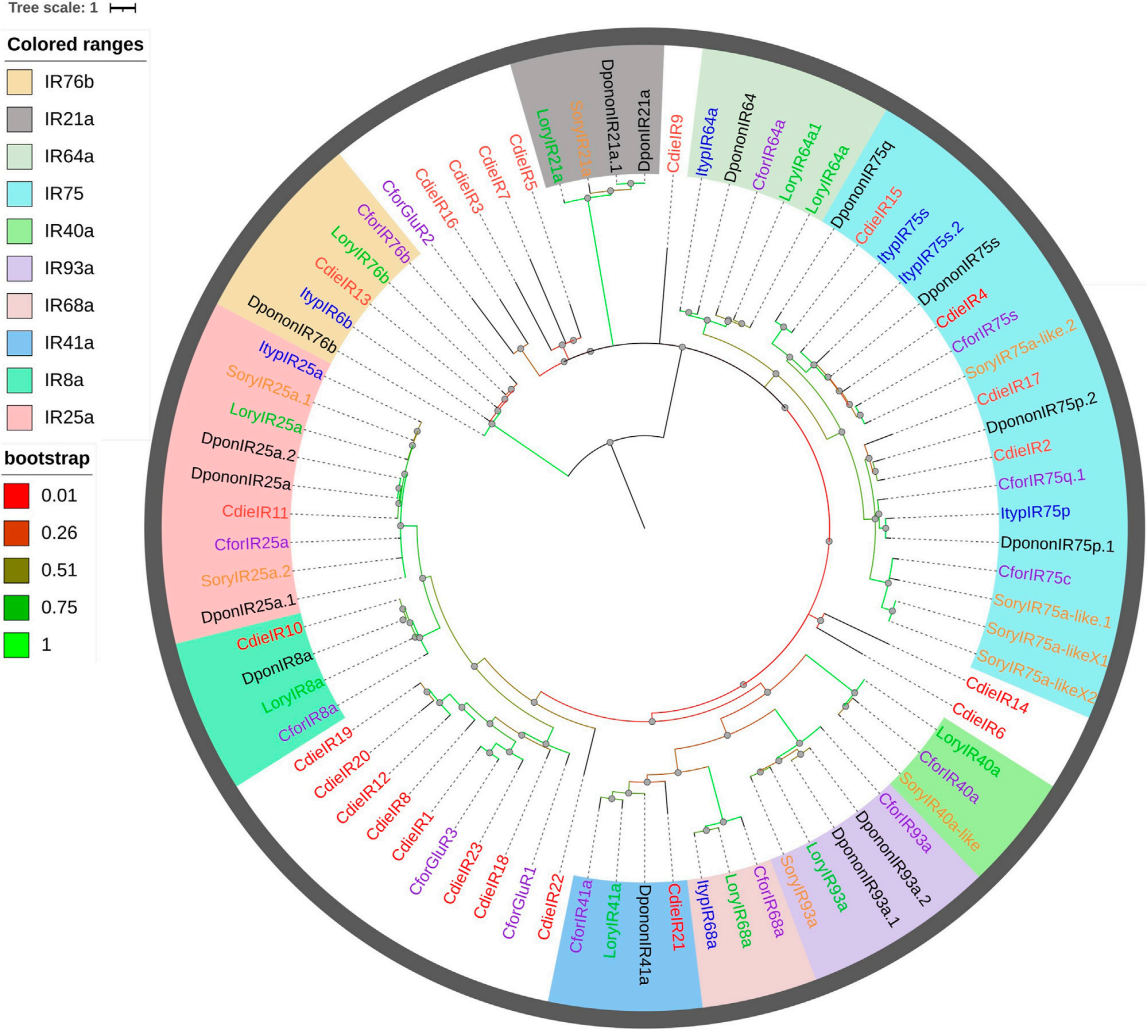
Maximum likelihood phylogenetic tree of candidate IRs in *C. dieckmanni* (Cdie, red), *Dendroctonus ponderosae* (Dpon, black), *Ips typographus* (Ityp, blue), *Lissorhoptrus oryzophilus* (Lory, green), *Sitophilus oryzae* (Sory, orange), and *Cylas formicarius* (Cfor, purple). The scale bar represents 1 amino acid substitution per site.

We identified 23 IRs from *C. dieckmanni* antennae transcriptomes. A phylogenetic tree was built along with the IRs identified in other Curculionoidea species (*Cylas formicarius*, *D. ponderosae*, *Ips typographus*, *L. oryzophilus*, and *S. oryzae*; Figure 7). Phylogenetic analysis showed that CdieIR2, CdieIR4, CdieIR15, and CdieIR17 were clustered in IR75; CdieIR10 clustered in IR8a; CdieIR11 clustered in IR25a; CdieIR13 clustered in IR76b; and CdieIR21 clustered in IR41a. These were highly conserved. Related genes were identified based on other Curculionoidea species. The genes encoding nine IRs (CdieIR1, CdieIR8, CdieIR11, CdieIR12, CdieIR13, CdieIR19, CdieIR20, CdieIR22, and CdieIR23) had ORFs similar to or longer than 1,600 bp, and the ORF of CdieIR7 gene was 1,431 bp. The lengths of these nine IRs were comparable to the full lengths of such genes, and the genes of the remaining 14 IRs (CdieIR2, CdieIR3, CdieIR4, CdieIR5, CdieIR6, CdieIR7, CdieIR9, CdieIR10, CdieIR14, CdieIR15, CdieIR16, CdieIR17, CdieIR18, and CdieIR21) had partial ORFs of such genes. The number of transmembrane domains of the 23 IRs ranged from one to six (Supplementary Table S2.5).

### Gustatory Receptors

**FIGURE 8:**
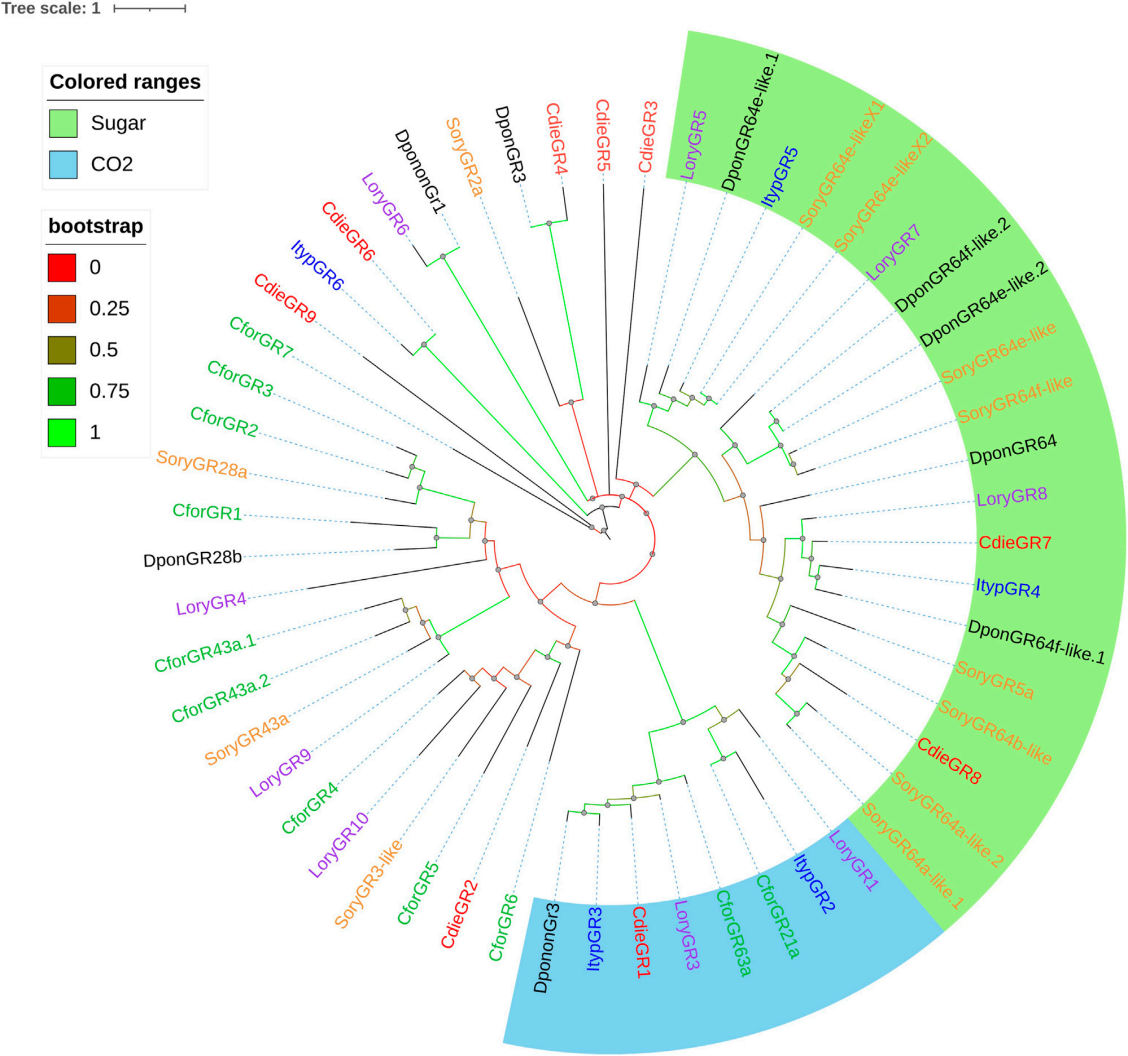
Maximum likelihood phylogenetic tree of candidate GRs in *C. dieckmanni* (Cdie, red), *Dendroctonus ponderosae* (Dpon, black), *Lissorhoptrus oryzophilus* (Lory, purple), *Ips typographus* (Ityp, blue), and *Cylas formicarius* (Cfor, green). The scale bar represents the 1 amino acid substitutions per site.

From the transcriptome data, we identified 9 GR candidate transcripts with 1–11 transmembrane domains and homology ranging from 29.16 to 61.07%. The genes of 5 GRs (CdieGR1, CdieGR5, CdieGR6, CdieGR8, and CdieGR9) had ORFs longer than 1,000 bp (Supplementary Table S2.6). A phylogenetic tree was constructed to analyze the functional classification of GRs in C. dieckmanni transcriptomes based on the GRs identified in other Curculionoidea species (*C. formicarius*, *D. ponderosae*, *I. typographus*, *L. oryzophilus*, and *S. oryzae*; Figure 8). Phylogenetic analysis showed that three GRs (CdieGR4, CdieGR7, and CdieGR8) clustered in the gustatory receptor for sugar receptors, while CdieGR1 clustered in the gustatory receptor for CO_2_ receptors.

### RPKM Analysis of DEGs and qRT-PCR Verification

**FIGURE 9:**
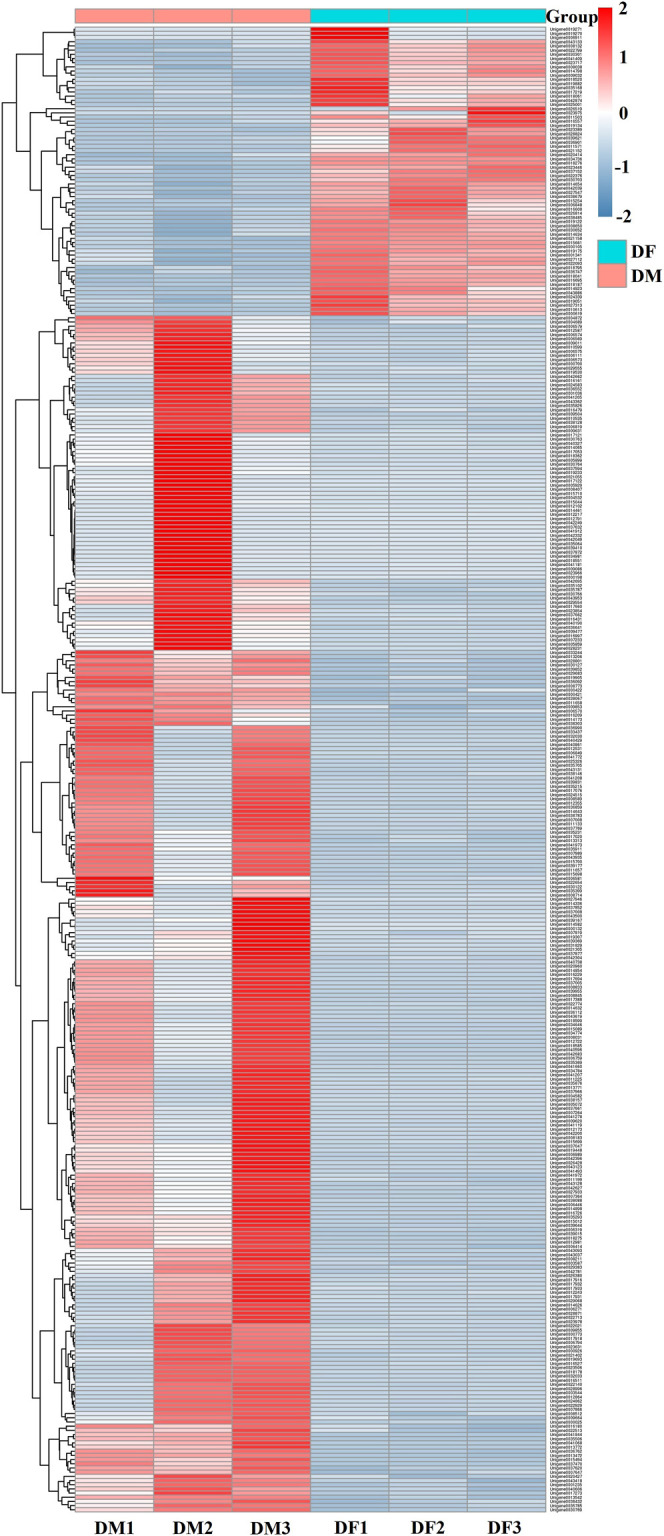
Heatmap of significant differentially expressed genes in *C. dieckmanni*. Cluster analyses were based on log2RPKM. Each column represents a sample and each row represents a gene. Red boxes represent highly expressed genes. Blue boxes represent lowly expressed genes. DM1, DM2, and DM3 represent three replicate samples of males. DF1, DF2, and DF3 represent three replicate samples of females.

The RPKM analysis showed that a total of 355 genes were differentially expressed in male and female antennae, among which, 69 were significantly male-biased and 286 were significantly female-biased (Figure 9, Supplementary Tables S3, S4), including only three chemosensory genes. Two candidate odorant-binding proteins (CdieOBP19 and CdieOBP20) were expressed at significantly higher levels in female antennae than in male antennae (FDR < 0.05) (Supplementary Table S4). One candidate odorant receptor protein (CdieOR13) was expressed at significantly higher levels in female antennae than in male antennae (FDR < 0.05) (Supplementary Table S4).

**FIGURE 10:**
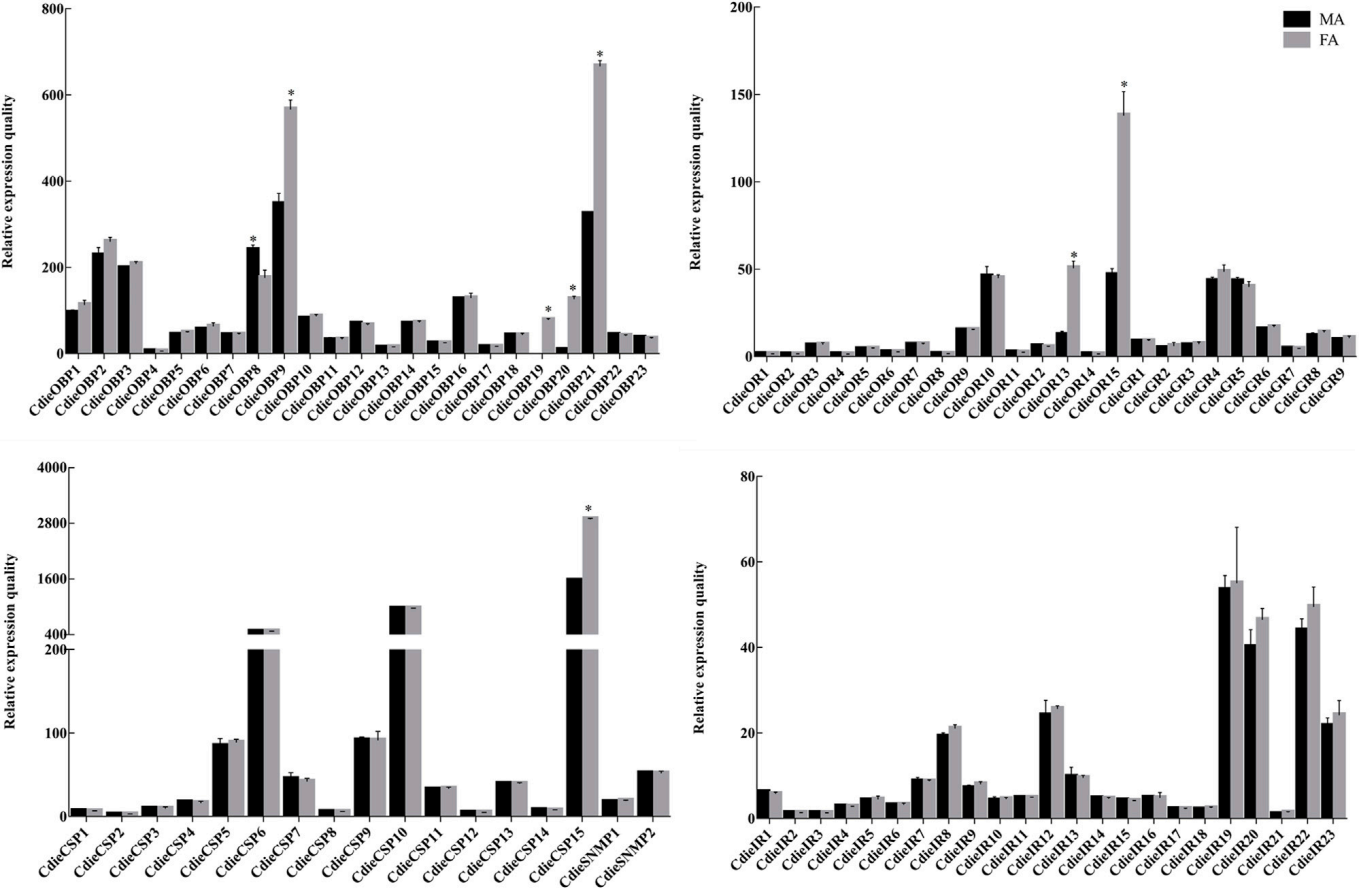
Relative expression levels of chemosensory genes in *C. dieckmanni* OBPs. FA: Female antennae; MA: Male antennae. The asterisks above each bar represent significant differences (*p* < 0.05).

To validate and further understand the functions of the chemosensory genes in adult female and male *C. dieckmanni* antennae, we studied the expression profiles of these genes using qRT-PCR. The primers are listed in Supplementary Table S7. The qRT-PCR results showed that all chemosensory genes were expressed in both male and female antennae, except CdieOBP19, which was only expressed in female antennae. Three OBPs (CdieOBP9, CdieOBP20, and CdieOBP21) were significantly female-biased, one OBP (CdieOBP8) was significantly male-biased, one CSP (CdieCSP15) was significantly female-biased, two ORs (CdieOR13 and CdieOR15) were significantly female-biased, and no SNMPs, IRs, or GRs showed differential expression between males and females (Figure 10).

## Discussion

Chemosensory proteins play essential roles in insect survival and reproduction and, thus, have been studied in several insect species (Guo et al., 2011). The present study contributes to build up our knowledge on the functioning of olfactory genes in the Curculionidae family and, specifically, constitutes a foundation for understanding the olfactory mechanisms of *C. dieckmanni*, a pest that affect hazelnut crops. Our results provide new directions for safer and less toxic methods to control *C. dieckmanni* populations.

Transcriptome analysis using BLASTx identified 87 chemosensory genes, including OBPs, CSPs, SNMPs, ORs, IRs, and GRs, from the antennae of male and female *C. dieckmanni*. These results enriched the chemosensory genes of *Curculionidae*. OBPs and CSPs are involved in the first step of hydrophobic odorant recognition, and their identification is a prerequisite for exploring the olfactory mechanisms of *C. dieckmanni*.

The numbers of OBPs and CSPs identified in *C. dieckmanni* were 23 and 15, respectively, which were lower than the OBPs (49) and CSPs (20) of *T. castaneum* but were similar to the OBPs (24) and CSPs (12) of *Anthonomus grandis* (Paula et al., 2018). We found that 11 were typical OBPs, 12 were Minus-C OBPs, with the OBPs from five *Curculionoidea* species (*D. adjunctus*, *D. armandi*, *L. oryzophilus*, *S. zeamais*, and *T. yunnanensis*), which indicated that they may serve the same functions and have the same ancestor gene. The qRT-PCR results showed that the expression levels of CdieOBP9, CdieOBP20, and CdieOBP21 in female antennae were significantly higher than in male antennae, and CdieOBP19 was only expressed in female antennae, indicating that these genes may play the role of binding sex pheromones. Odorant-binding proteins are the main carriers of chemical signals as aggregation, alarm, and sex pheromones. ApisOBP3 and ApisOBP7 in adults and nymphs of *A. pisum* carry the alarm pheromone (E)-β-farnesene (Zhang et al., 2017). Most weevil sex pheromones are released by males, such as boll weevils and pecan weevils, the sex pheromones of boll weevils are alpha-pinene, limonene, beta-caryophyllene, and beta-bisabolol (Tumlinson et al., 1969). The sex pheromones of pecan weevils are delta3-21Hy, deta1,3-22Hy, delta1-23Hy, and delta1-24Hy (Hedin et al., 1979). Because CdieOBP9, CdieOBP20, and CdieOBP21 are also expressed in males, it is speculated that they may function as pheromone carriers for male sexual behavior. OBPs showed an average of 58.26% homology with other insects, while the homology value for CSPs reached 70.83%. The reason for this discrepancy is that, as compared with OBPs, CSPs have four highly conserved cysteines and lower binding specificity but were more flexible in recognizing different ligands (Sánchez-Gracia et al., 2009; Pelosi et al., 2018). We found that one CSP (CdieCSP15) was highly expressed in female antennae; The function of this CSP requires further investigation.

Moreover, because SNMPs were discovered in ORNs, they may play important roles in insect olfactory recognition (Nichols and Vogt, 2008). SNMP1 and SNMP2 were identified in *C. dieckmanni* transcriptomes and in other insect species (Hildebbrand, 1996). Hylogenetic analysis showed that CdieSNMP1 and CdieSNMP2 clustered with the SNMP1 and SNMP2 of other *Curculionoidea* species (*S. zeamais*, *I. typographus*, *C. formicarius*, and *D. ponderosae*) and were most closely related to *D. ponderosae*. The RPKM and qRT-PCR results showed that no SNMPs were differentially expressed between males and females.

Olfactory sensilla in insect antennae are controlled by two or more ORNs, which express a range of chemosensory receptor protein families and are involved in the detection and transduction of odorant signals (Benton et al., 2006; Sato and Touhara, 2009). These proteins mainly include ORs, IRs, and GRs (Benton et al., 2009; Koenig et al., 2015). Our study identified a total of 15 ORs in *C. dieckmanni* transcriptomes, which was lower than that of *D. ponderosae* and *I. typographus* (49 and 43) (Andersson et al., 2013). The difference in the number of OR genes may be a result of the method and depth of sequencing. By comparing the complete amino acid sequences, we identified the main OR subfamily and ORco genes from *D. ponderosae*, *I. typographus*, *C. formicarius*, and *S. oryzae*. Phylogenetic analyses showed that 11 ORs of *C. dieckmanni* (except CdieOR1, CdieOR3, and CdieOR4) were contained in six groups (1, 2, 3, 4, 5, and 7, with no CdieORs in group 6 of the nine beetle groups) (Mitchell et al., 2020), which is consistent with other *Curculionoidea* species, including *D. ponderosae*, *I. typographus*, *C. formicarius*, and *S. oryzae* (Andersson et al., 2013; Bin et al., 2017). One OR (CdieOR15) clustered in a branch with the highly conserved Orco of *D. Ponderosae*, *C. formicarius*, and *S. oryzae*. They may serve the same role in odorant detection. The qRT-PCR results showed that two ORs (CdieOR13 and CdieOR15) were significantly female-biased. CdieOR15 may serve a different function in males and females when detecting specific odorants, for example when a female is looking for a male or for a place to lay eggs (Jacquin-Joly and Merlin, 2004; Benton et al., 2006; Sato and Touhara 2009). CdieOR13 clustered on the branch of group 7 together with DponOR33, DponOR34, and ItypOR33. It may play a role in the female detection of sex pheromones released from males (Antony et al., 2021; Yuvaraj et al., 2021). This needs to be verified by further ligand binding studies.

IRs are involved in odorant detection (Benton et al., 2009; Croset et al., 2010; Abuin et al., 2011). CdieIR10 clustered in the IR8a branch, CdieIR11 clustered in the IR25a branch, and IR8a and IR25a are co-expressed with IRs and essential for the functioning of IRs (Benton et al., 2009; Rytz et al., 2013; Joseph and Carlson, 2015; Fleischer et al., 2018). CdieIR2 clustered in IR75p, CdieIR4 clustered in IR75s, CdieIR13 clustered in IR76b, CdieIR15 clustered in IR76q, CdieIR17 clustered in IR75c, and CdieIR21 clustered in IR41a. These results may suggest that IRs of the same branch may play the same role in the antennae. GRs play key roles in detecting gustatory chemicals that govern insect feeding, courtship, mating, and the location of spawning sites (Freeman et al., 2014; Sollai et al., 2018). We identified 9 GRs in *C. dieckmanni* antennae transcriptomes. This number was lower than the number of ORs, presumably because antennae are not major gustatory organs. The phylogenetic tree showed that CdieGR1 clustered in the CO_2_ branch and may be involved in CO_2_ detection. Interestingly, CdieGR4, CdieGR7, and CdieGR8 clustered in the sugar branch, and presumably play a role in sugar detection. CdieGR4 and DponGR3 clustered in the same branch, CdieGR6 and ItypGR6 clustered in the same branch, CdieGR9 and CforGR7 clustered in the same branch, and CforGR4, CforGR5, CforGR6, LoryGR10, SoryGR3-like, and CdieGR2 clustered in the same branch. GRs on the same branch may have the same function.

In this study, a total of 44,024 unigenes were obtained from the antennae of female and male adults of *C. dieckmanni* using high-throughput sequencing. Our transcriptome analysis identified 87 olfactory genes in antennae, including 23 OBPs, 15 CSPs, 2 SNMPs, 15 ORs, 23 IRs, and 9 GRs. Male- and female-specific chemosensory genes are possibly involved in odorant detection and recognition, and their expression was validated by qRT-PCR. Among the chemosensory genes, we found significant differences in the expression of CdieOBP8, CdieOBP9, CdieOBP19, CdieOBP20, CdieOBP21, CdieCSP15, CdieOR13, and CdieOR15 between adult male and female *C. dieckmanni*. These results add to the known chemosensory genes of Curculionidae and contribute to further understand the olfactory and gustatory mechanisms of *C. dieckmanni*. Our results may provide new insights for the control of this pest.

## Data Availability

The datasets presented in this study can be found in online repositories. The names of the repository/repositories and accession number(s) can be found below: https://www.ncbi.nlm.nih.gov/, PRJNA769532.
