# Supplementary material for: Transcriptome Analysis of Antennal Chemosensory Genes in Curculio Dieckmanni Faust. (Coleoptera: Curculionidae)
Source: Front Physiol. 2022 May 9;13:896793. doi: 10.3389/fphys.2022.896793 (PMC9124802; doi:10.3389/fphys.2022.896793)
Supplement: Supplementary file 1 [file DataSheet2.PDF]

Supplementary Material

Supplementary Tables（Table S1.1_S2.6）

Table S1.1. Information of the three samples used for transcriptome analysis.

Sample	Read number		Low quality (%)	Adapter (%)	GC (%)	Q20(%)	Q30(%)
	Before filter	After filter					
DM1	57,410,056	55,983,482(97.52%)	1,159,334 (2.02%)	266,858 (0.46%)	43.21	98.57%	95.65%
DM2	50,659,328	49,406,426(97.53%)	1,013,364 (2%)	239,196 (0.47%)	42.81	98.59%	95.72%
DM3	43,093,924	41,989,468(97.44%)	874,334 (2.03%)	229,830 (0.53%)	43.29	98.57%	95.67%
DF1	50,324,912	49,095,002(97.56%)	990,130 (1.97%)	239,428 (0.48%)	43.10	98.59%	95.72%
DF2	46,008,558	44,906,190(97.60%)	919,034 (2%)	182,988 (0.4%)	43.10	98.59%	95.70%
DF3	50,345,942	49,073,722(97.47%)	1,063,588 (2.11%)	208,252 (0.41%)	43.00	98.54%	95.60%

Table S1.2. Summary of transcriptome assembly.

Statistics project	Number
Total assembled bases	43,060,197
Genes number	44,024
Genes number of DF	42,971
Genes number of DM	43,190
GC percentage	37.98%
N50 of unigenes (nt)	1861
Min length of unigenes (nt)	201
Average length of unigenes (nt)	978
Max length of unigenes (nt)	27,588

Table S2.1. Information for OBPs in *C. dieckmanni.*

Gene name	Unigene ID	OFR(AA)	Complete ORF	Full length	Signal peptide	Blastx annotation					
						Name	ACC.NO.	species	score	E-value	Identity
CdieOBP1	Unigene0033737	429(142)	yes	952	1-29	odorant binding protein 11	*AMP19493.1*	*Tomicus yunnanensis*	155	2e-45	55.32%
CdieOBP2	Unigene0035042	405(134)	yes	407	1-23	odorant binding protein 5	ALM64967.1	*Dendroctonus armandi*	134	3e-37	54.87%
CdieOBP3	Unigene0036587	330(109)	Yes	520	1-16	odorant binding protein	AHE13800.1	*Lissorhoptrus oryzophilus*	174	2e-52	53.62%
CdieOBP4	Unigene0038578	375(124)	yes	406	1-25	odorant-binding protein 24	QKV35001.1	*Dendroctonus adjunctus*	129	2e-35	49.56%
CdieOBP5	Unigene0041945	402(133)	yes	497	1-27	odorant binding protein 4	AKK25132.1	*Dendroctonus ponderosae*	200	4e-63	77.42%
CdieOBP6	Unigene0042556	432(143)	yes	545	1-26	Odoran binding protein 33	JAA74401.1	*Pagiophloeus tsushimanus*	218	1e-69	77.69%
CdieOBP7	Unigene0042870	366(121)	yes	475	1-23	genernal odorant binding protein 83a	XP_030746449.1	*Sitophilus oryzae*	229	3e-74	75.18%
CdieOBP8	Unigene0042915	423(140)	yes	532	1-26	odorant binding protein 13	ALM64971.1	*Dendroctonus armandi*	148	2e-42	47.33%
CdieOBP9	Unigene0003257	405(134)	yes	2349	ND	odorant binding protein 19	AKK25143.1	*Dendroctonus ponderosae*	156	2e-40	65.45%
CdieOBP10	Unigene0009884	408(135)	yes	488	1-31	odorant binding protein	AHE13799.1	*Lissorhoptrus oryzophilus*	148	1e-42	49.26%
CdieOBP11	Unigene0011143	417(138)	yes	559	ND	odorant binding protein 4	ALM64966.1	*Dendroctonus armandi*	204	2e-64	78.05%
CdieOBP12	Unigene0013319	423(140)	yes	478	1-23	odorant-binding protein 23	QKV35000.1	*Dendroctonus adjunctus*	183	3e-56	59.85%
CdieOBP13	Unigene0013657	474(157)	yes	486	1-28	odorant-binding protein 11b	QKV34990.1	*Dendroctonus adjunctus*	229	2e-74	74.26%
CdieOBP14	Unigene0014126	375(124)	yes	448	1-21	odorant binding protein 8	ALM64970.1	*Dendroctonus armandi*	78.2	4e-15	37.86%
CdieOBP15	Unigene0014127	417(138)	yes	433	1-20	odorant binding protein	QFO46770.1	*Cylas formicarius*	77.8	5e-15	37.40%
CdieOBP16	Unigene0014436	411(136)	yes	530	1-20	odorant binding protein 6	AVI04887.1	*Anthonomus grandis*	213	4e-68	69.85%
CdieOBP17	Unigene0016601	396(131)	yes	452	1-20	odorant-binding protein 1	AMP19483.1	*Tomicus yunnanensis*	120	8e-32	42.86%
CdieOBP18	Unigene0023969	339(112)	Yes	689	ND	odorant binding protein 24	QKV35001.1	*Dendroctonus adjunctus*	108	2e-26	43.24%
CdieOBP19	Unigene0023975	402(133)	yes	740	ND	Odorant binding protein 14	ALM64972.1	*Dendroctonus armandi*	182	6e-55	64.66%
CdieOBP20	Unigene0028824	468(155)	yes	567	1-19	Odorant binding protein 10	AVI04891.1	*Anthonomus grandis*	144	5e-40	50.00%
CdieOBP21	Unigene0013317	438(145)	yes	510	1-22	odorant binding protein	AHE13793.1	*Lissorhoptrus oryzophilus*	231	5e-75	77.30%
CdieOBP22	Unigene0013929	456(151)	yes	492	ND	odorant binding protein 39	QCT83293.1	*Sitophilus zeamais*	155	3e-45	53.42%
CdieOBP23	Unigene0019314	333(110)	no	621	ND	odorant binding protein 9	AKK25135.1	*Dendroctonus ponderosae*	129	1e-34	45.52%

Table S2.2. Information for CSPs in *C. dieckmanni.*

Gene name	Unigene ID	OFR(AA)	Complete ORF	Full length	Signal peptide	Blastx annotation					
						Name	ACC.NO.	species	score	E-value	Identity
CdieCSP1	Unigene0035589	354(117)	no	437	ND	chemosensory protein 4	AXF54072.1	Dendroctonus armandi	189	5e-59	74.56%
CdieCSP2	Unigene0036010	231(76)	no	335	ND	Putative chemosensory protein 4	AVI04875.1	Anthonomus grandis	188	4e-59	76.58%
CdieCSP3	Unigene0036864	360(119)	yes	383	1-28	Putative chemosensory protein 6	AVI04877.1	Anthonomus grandis	174	3e-53	61.48%
CdieCSP4	Unigene0038516	294(97)	no	307	ND	chemosensory protein 6	AKK25149.1	Dendroctonus ponderosae	145	8e-43	75.51%
CdieCSP5	Unigene0043379	441(146)	no	556	ND	chemosensory protein 4	AMP19499.1	Tomicus yunnanensis	205	1e-64	68.49%
CdieCSP6	Unigene0005886	393(130)	no	474	1-24	chemosensory protein 9	AHE13804.1	Lissorhoptrus oryzophilus	175	3e-53	63.91%
CdieCSP7	Unigene0007660	378(125)	no	394	1-23	Putative chemosensory protein 4	AVI04875.1	Anthonomus grandis	205	9e-66	74.60%
CdieCSP8	Unigene0000694	963(320)	no	2472	ND	chemosensory protein 4	QPZ89238.1	*Dendroctonus adjunctus*	333	9e-105	55.59%
CdieCSP9	Unigene0009732	234(77)	yes	362	1-16	chemosensory protein 1	AXF54070.1	Dendroctonus armandi	179	1e-55	76.70%
CdieCSP10	Unigene0000791	384(127)	yes	844	ND	chemosensory protein 8	AXF54075.1	Dendroctonus armandi	223	1e-70	82.68%
CdieCSP11	Unigene0012300	423(140)	yes	667	1-28	chemosensory protein 1	AMP19496.1	*Tomicus yunnanensis*	234	1e-75	85.48%
CdieCSP12	Unigene0013800	369(122)	yes	633	ND	chemosensory protein 9	QPZ89241.1	*Dendroctonus adjunctus*	190	9e-59	75.42%
CdieCSP13	Unigene0015162	366(121)	yes	533	ND	chemosensory protein 2	AXF53965.1	Dendroctonus armandi	184	1e-56	64.46%
CdieCSP14	Unigene0015360	387(128)	yes	405	1-19	chemosensory protein 5	AIX97045.1	Monochamus alternatus	104	7e-26	44.53%
CdieCSP15	Unigene0016846	387(128)	yes	506	1-28	chemosensory protein 3	AMP19498.1	Tomicus yunnanensis	194	6e-61	82.41%

Table S2.3. Information for SNMPs in *C. dieckmanni.*

Gene name	Unigene ID	OFR(AA)	Complete ORF	Full length	TMD	Blastx annotation					
						Name	ACC. NO.	Species	score	E-value	Identity
CdieSNMP1a	Unigene0028918	1695(564)	yes	2898	6	sensory neuron membrane protein 1a	AGI05171.1	Dendroctonus ponderosae	830	0	74.04%
CdieSNMP2	Unigene0031440	1575(524)	yes	1780	3	sensory neuron membrane protein 2	AGI05184.1	Dendroctonus ponderosae	632	0	56.84%

Table S2.4. Information for ORs in *C. dieckmanni*.

Gene name	Unigene ID	OFR(AA)	Complete ORF	Full length	TMD	Blastx annotation					
						Name	ACC.NO.	Species	score	E-value	Identity
CdieOR1	Unigene0039270	552(183)	Yes	571	3	odorant receptor 33	QXE93214.1	Eucryptorrhynchus scrobiculatus	129	6e-32	36.02%
CdieOR2	Unigene0040449	347(114)	yes	354	2	odorant receptor 32	QXE93258.1	Eucryptorrhynchus brandti	120	1e-29	50.86%
CdieOR3	Unigene0041987	105(34)	yes	438	1	odorant receptor 41	QXE93221.1	Eucryptorrhynchus scrobiculatus	176	3e-50	54.67%
CdieOR4	Unigene0042084	78(25)	yes	301	1	odorant receptor Or1	KOC67813.1	Habropoda laboriosa	61.6	2e-08	42.27%
CdieOR5	Unigene0042161	276(91)	yes	277	1	PREDICTED: odorant receptor Or2-like	XP_019761329.1	Dendroctonus ponderosae	89	1e-19	44.57%
CdieOR6	Unigene0043479	239(78)	yes	260	1	odorant receptor 49b-like	XP_030767718.1	Sitophilus oryzae	126	3e-33	66.28%
CdieOR7	Unigene0005727	175(57)	yes	357	2	PREDICTED: odorant receptor 47b-like	XP_019755291.1	Dendroctonus ponderosae	102	3e-23	40.17%
CdieOR8	Unigene0006375	195(64)	yes	339	2	odorant receptor 17	QXE93243.1	Eucryptorrhynchus brandti	167	2e-47	67.89%
CdieOR9	Unigene0000605	534(177)	yes	2251	1	odorant receptor 44	QXE93223.1	Eucryptorrhynchus scrobiculatus	159	8e-42	60.15%
CdieOR10	Unigene0008849	1125(374)	yes	1436	7	odorant receptor 23	QXE93249.1	Eucryptorrhynchus brandti	655	0	81.32%
CdieOR11	Unigene0010909	105(34)	yes	244	1	odorant receptor 49b-like	XP_030767718.1	Sitophilus oryzae	72.8	7e-13	41.25%
CdieOR12	Unigene0015304	387(128)	yes	799	4	odorant receptor 3	QXE93184.1	Eucryptorrhynchus scrobiculatus	398	4e-135	68.80%
CdieOR13	Unigene0019134	1220(405)	yes	1228	6	odorant receptor 21	QXE93247.1	Eucryptorrhynchus brandti	598	0	69.67%
CdieOR14	Unigene0027868	136(44)	yes	295	2	odorant receptor 31	QXE93257.1	Eucryptorrhynchus brandti	113	1e-28	54.64%
CdieOR15	Unigene0008561	1449(482)	yes	1959	9	odorant receptor 24	QXE93205.1	Eucryptorrhynchus scrobiculatus	919	0	94.40%

Table S2.5. Information for IRs in *C. dieckmanni.*

Gene name	Unigene ID	OFR(AA)	Complete ORF	Full length	TMD	Blastx annotation					
						Name	ACC.NO.	Species	score	E-value	Identity
*Cdie*IR1	Unigene0000235	2754(917)	yes	2875	4	ionotropic receptor	AUF73087.1	Anoplophora chinensis	1020	0	53.67%
*Cdie*IR2	Unigene0035173	144(47)	no	209	1	ionotropic receptor 75a-like isoform X3	XP_045473000.1	Harmonia axyridis	45.1	0.004	50.98%
*Cdie*IR3	Unigene0037081	96(31)	yes	320	1	ionotropic receptor 25a	XP_030756779.1	Sitophilus oryzae	135	3e-34	76.92%
*Cdie*IR4	Unigene0041048	186(61)	no	251	1	ionotropic receptor 1	QNH68025.1	Apriona germari	85.5	4e-17	51.76%
*Cdie*IR5	Unigene0041105	78(25)	yes	224	1	ionotropic receptor 75a-like	XP_030768286.1	Sitophilus oryzae	126	7e-32	79.17%
*Cdie*IR6	Unigene0043539	279(92)	no	290	1	ionotropic receptor 4	AVH87292.1	Holotrichia parallela	68.6	7e-11	37.23%
*Cdie*IR7	Unigene0006217	1431(476)	no	1758	3	Ionotropic receptor 873	PSN54294.1	Blattella germanica	142	8e-32	28.43%
*Cdie*IR8	Unigene0007574	2652(883)	no	2835	3	ionotropic receptor	AUF73077.1	Anoplophora chinensis	1070	0	56.96%
*Cdie*IR9	Unigene0007713	207(68)	no	703	1	ionotropic receptor 8a	AGI05169.1	Dendroctonus ponderosae	306	2e-94	58.55%
*Cdie*IR10	Unigene0007907	990(329)	yes	1037	3	ionotropic receptor 8a	AGI05169.1	Dendroctonus ponderosae	702	0	96.52%
*Cdie*IR11	Unigene0008017	2043(680)	no	2067	2	PREDICTED: ionotropic receptor 25a	XP_019763174.1	Dendroctonus ponderosae	1227	0	86.36%
*Cdie*IR12	Unigene0008225	2715(904)	yes	3092	6	ionotropic receptor	AUF73078.1	Anoplophora chinensis	809	0	46.49%
*Cdie*IR13	Unigene0009153	1722(573)	no	2149	5	ionotropic receptor 2	AVN97884.1	Anoplophora chinensis	576	0	52.43%
*Cdie*IR14	Unigene0011536	1059(352)	no	1230	4	Ionotropic receptor 875	PSN31720.1	Blattella germanica	73.9	5e-10	22.56%
*Cdie*IR15	Unigene0011983	855(284)	yes	864	2	ionotropic receptor 1	APC94256.1	Pyrrhalta maculicollis	258	3e-79	46.49%
*Cdie*IR16	Unigene0011984	120(39)	yes	298	1	ionotropic receptor 75a-like	XP_044263055.1	Tribolium madens	92.8	2e-19	51.90%
*Cdie*IR17	Unigene0013256	276(91)	yes	471	1	Ionotropic receptor 75k	PSN49053.1	Blattella germanica	64.7	9e-09	36.47%
*Cdie*IR18	Unigene0014862	1047(348)	yes	1227	2	ionotropic receptor 7	AVH87295.1	Holotrichia parallela	654	0	86.76%
*Cdie*IR19	Unigene0018634	2838(945)	no	4139	6	ionotropic receptor	AUF73078.1	Anoplophora chinensis	749	0	46.93%
*Cdie*IR20	Unigene0019463	2100(699)	no	2913	4	ionotropic receptor	AUF73078.1	Anoplophora chinensis	629	0	45.92%
*Cdie*IR21	Unigene0020497	213(70)	yes	243	1	ionotropic receptor 21a-like	XP_026328722.1	Hyposmocoma kahamanoa	47.8	8e-04	28.57%
*Cdie*IR22	Unigene0022453	2646(881)	no	3289	4	ionotropic receptor 4	ANQ46496.1	Phyllotreta striolata	1539	0	87.44%
*Cdie*IR23	Unigene0024650	2622(873)	yes	2746	3	ionotropic receptor	AUF73087.1	Anoplophora chinensis	784	0	46.60%

Table S2.6. Information for GRs in *C. dieckmanni*.

Gene name	Unigene ID	OFR(AA)	Complete ORF	Full length	TMD	Blastx annotation					
						Name	ACC.NO.	Species	score	E-value	Identity
*Cdie*GR1	Unigene0021922	1401(466)	yes	1510	7	gustatory receptor 3	EFA04709.2	Tribolium castaneum	503	1e-170	61.07%
*Cdie*GR2	Unigene0043376	351(116)	no	451	1	gustatory receptor 14	APC94341.1	Pyrrhalta aenescens	59.7	4e-07	35.63%
*Cdie*GR3	Unigene0010694	1833(610)	no	2995	2	gustatory receptor 2	ALM26252.1	Athetis dissimilis	269	7.00e-74	29.16%
*Cdie*GR4	Unigene0013419	402(133)	no	634	1	gustatory receptor 3	AKK25150.1	Dendroctonus ponderosae	149	3e-42	60.90%
*Cdie*GR5	Unigene0015647	1128(375)	yes	1617	9	gustatory receptor 6	AVN97871.1	Anoplophora chinensis	448	5e-151	57.68%
*Cdie*GR6	Unigene0016755	1026(341)	yes	1633	11	putative gustatory receptor 10	AXY83426.1	Conopomorpha sinensis	378	3e-124	53.87%
*Cdie*GR7	Unigene0018105	1224(407)	no	1385	6	gustatory receptor for sugar taste 64f-like	XP_019760210.1	Dendroctonus ponderosae	422	2e-142	55.03%
*Cdie*GR8	Unigene0018802	756(251)	no	935	5	gustatory receptor for sugar taste 64a-like	XP_030761258.1	Sitophilus oryzae	226	2e-67	38.93%
*Cdie*GR9	Unigene0024547	420(139)	yes	1580	5	gustatory receptor 125	EFA07621.2	Tribolium castaneum	249	3e-70	43.60%
