# Supplementary material for: Transcriptome Analysis of Antennal Chemosensory Genes in Curculio Dieckmanni Faust. (Coleoptera: Curculionidae)
Source: Front Physiol. 2022 May 9;13:896793. doi: 10.3389/fphys.2022.896793 (PMC9124802; doi:10.3389/fphys.2022.896793)
Supplement: Supplementary file 4 [file DataSheet1.PDF]

# *Supplementary Material*

## Supplementary Figures

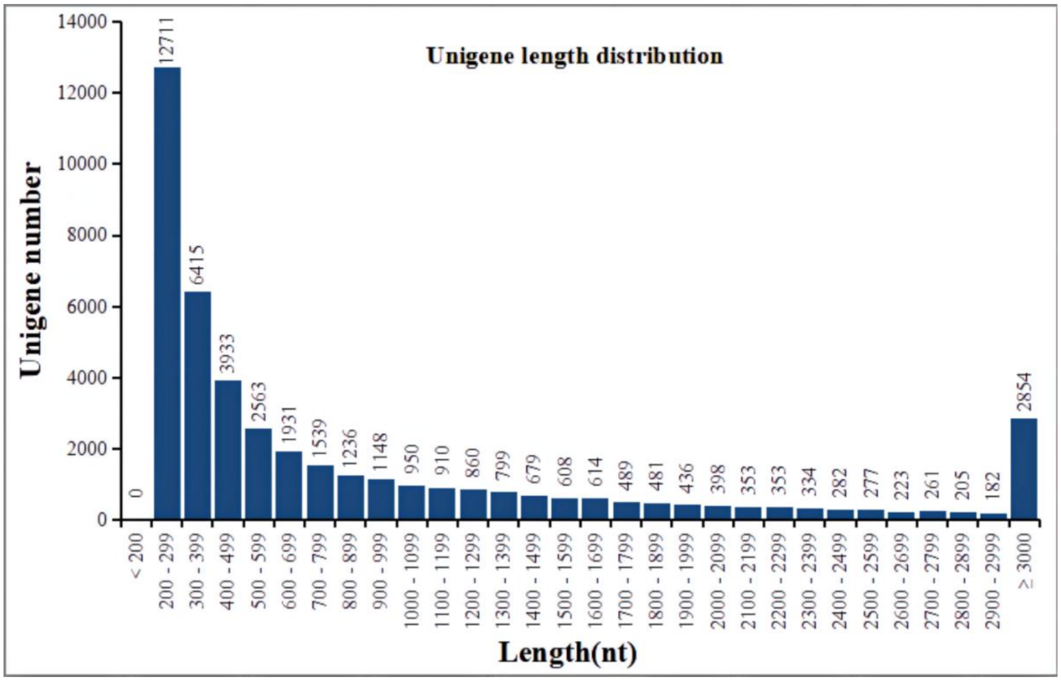


**Figure S1.** Length distribution of unigenes in *C. dieckmanni*. The histogram bars represent the numbers of unigenes in each length category.

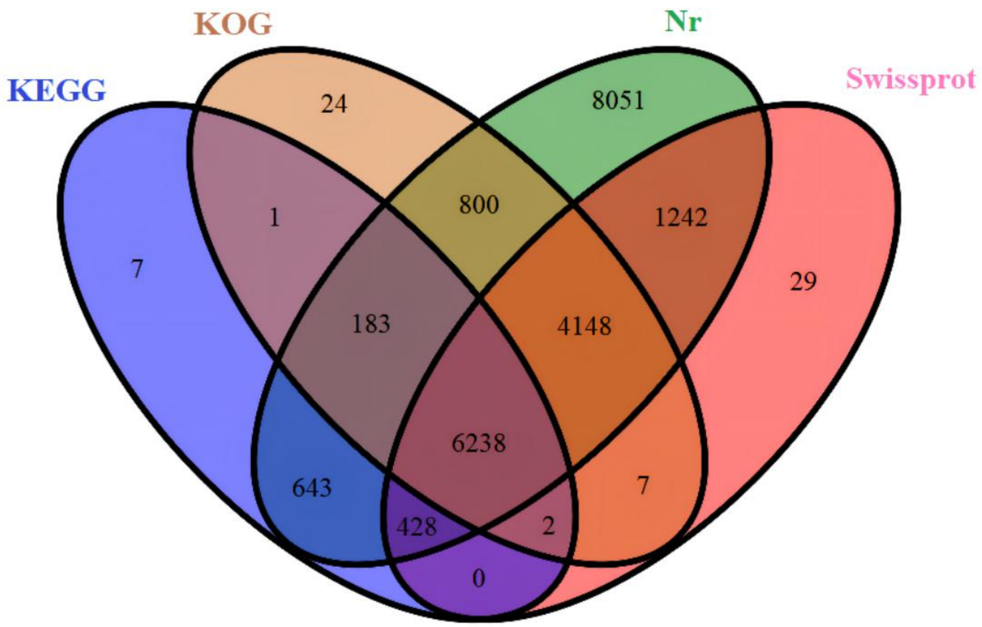


**Figure S2.** Venn diagrams of genes that were annotated in NR, Swiss-Port, KEGG and COG/KOG databases.

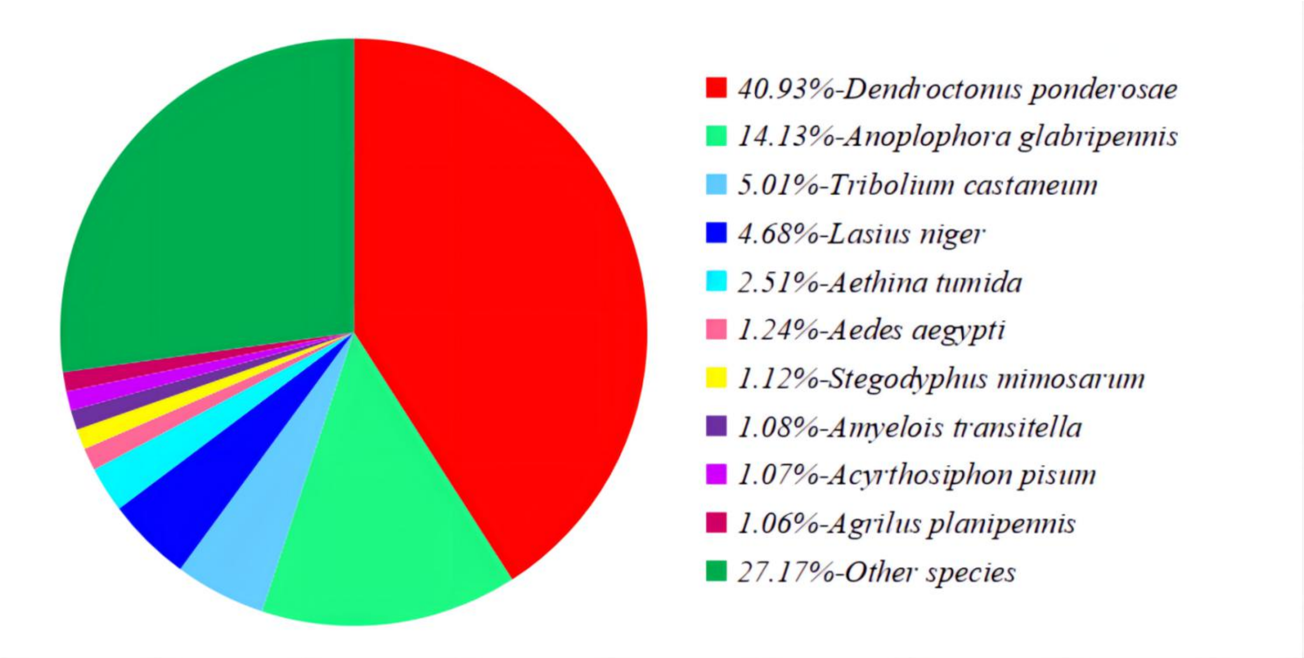


**Figure S3.** Species distribution of annotated genes in the Nr database.

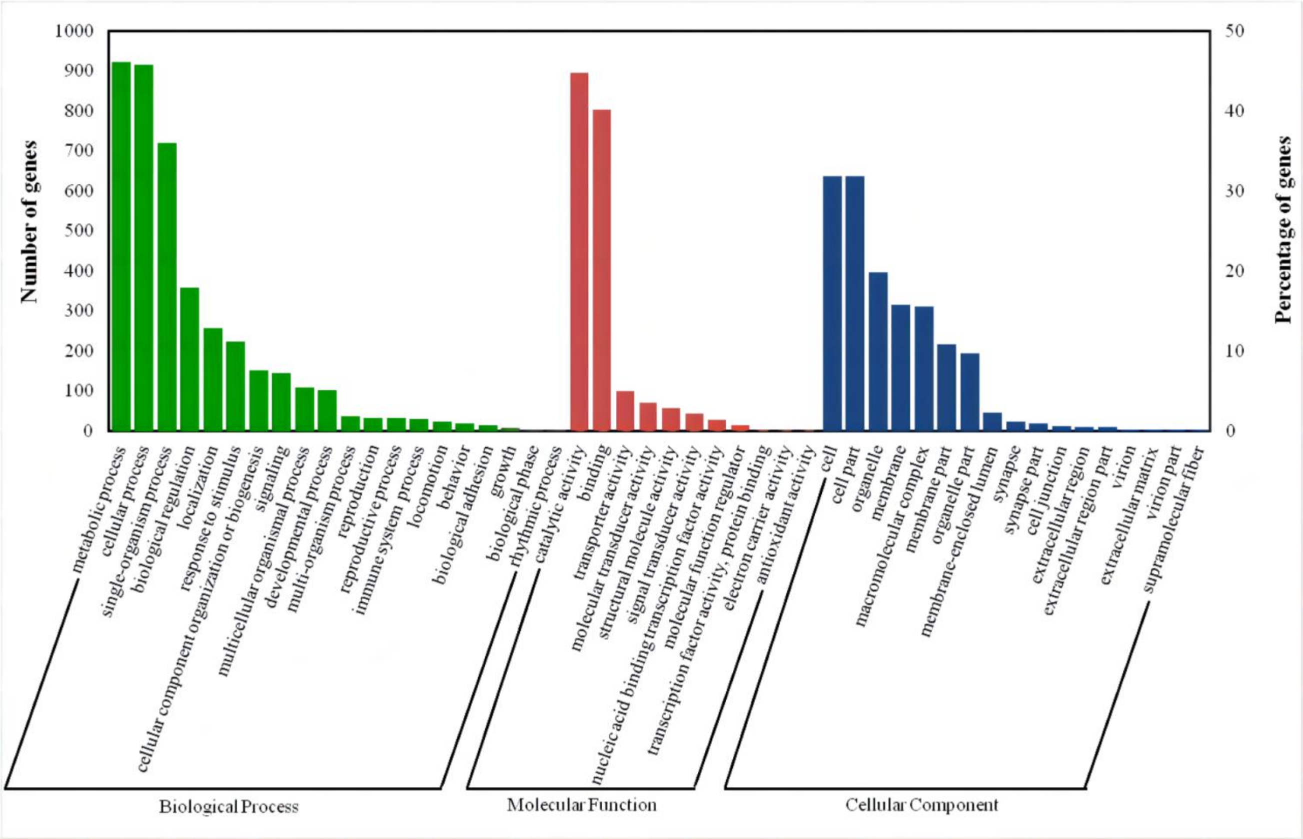


**Figure S4.** Gene Ontology (GO) classifications of *C. dieckmanni* unigenes.

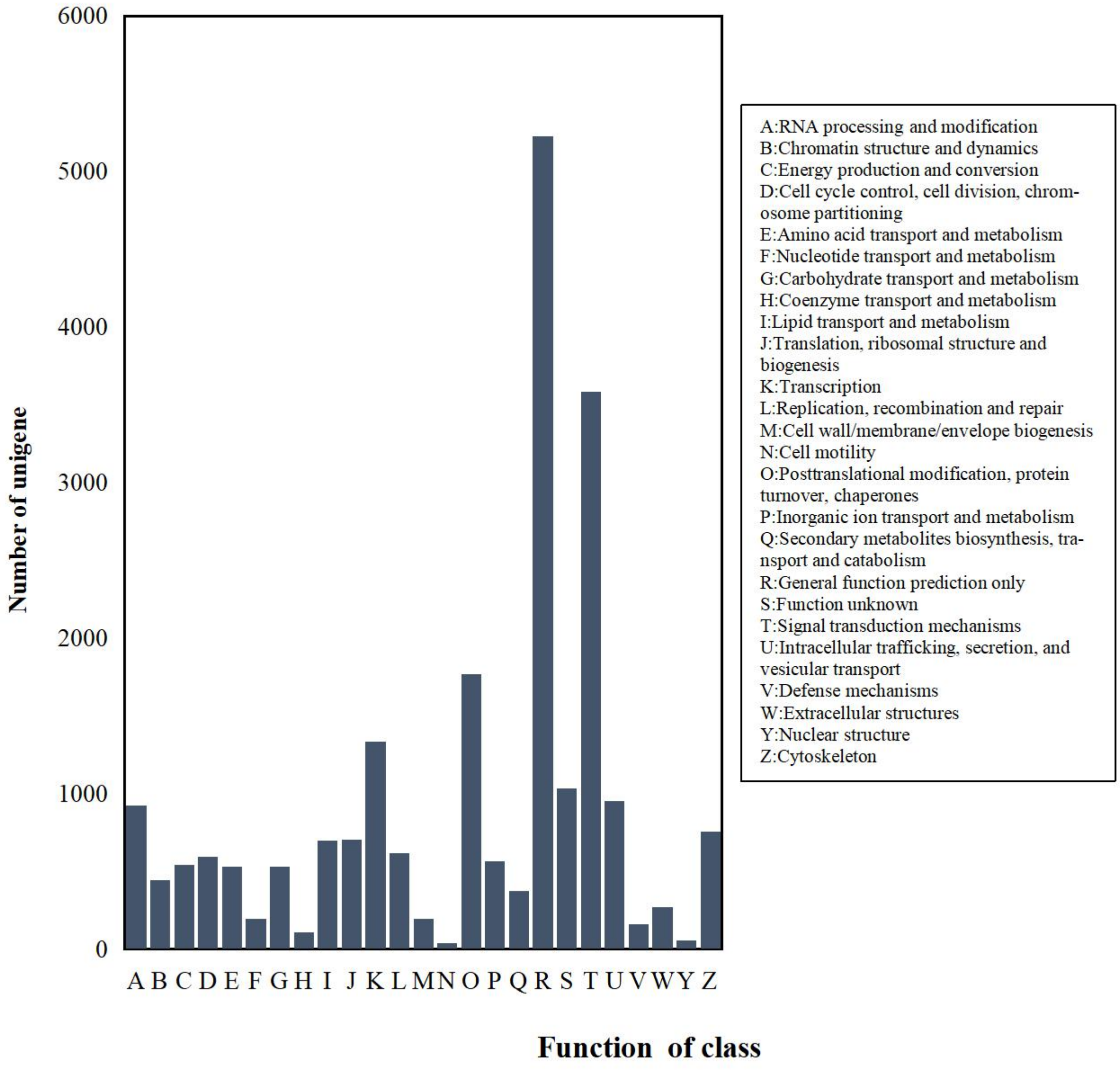


**Figure S5.** Karyotic Orthologous Groups classification of *C. dieckmanni* unigenes.
